# De novo *SCN2A* splice site mutation in a boy with Autism spectrum disorder

**DOI:** 10.1186/1471-2350-15-35

**Published:** 2014-03-20

**Authors:** Teresa Tavassoli, Alexander Kolevzon, A Ting Wang, Jocelyn Curchack-Lichtin, Danielle Halpern, Lily Schwartz, Sarah Soffes, Lauren Bush, David Grodberg, Guiqing Cai, Joseph D Buxbaum

**Affiliations:** 1 Seaver Autism Center for Research and Treatment, Icahn School of Medicine at Mount Sinai, New York, NY, USA; 2 Departments of Psychiatry, New York, NY, USA; 3 Departments of Pediatrics, New York, NY, USA; 4 Genetics and Genomic Sciences, and Neuroscience, New York, NY, USA; 5 Neuroscience, Icahn School of Medicine at Mount Sinai, New York, NY, USA; 6 Friedman Brain Institute, Icahn School of Medicine at Mount Sinai, New York, NY, USA; 7 Mindich Child Health and Development Institute, Icahn School of Medicine at Mount Sinai, New York, NY, USA

**Keywords:** DSM-5, autism spectrum disorder, *de novo SCN2A* splice site mutation

## Abstract

**Background:**

*SCN2A* is a gene that codes for the alpha subunit of voltage-gated, type II sodium channels, and is highly expressed in the brain. Sodium channel disruptions, such as mutations in *SCN2A*, may play an important role in psychiatric disorders. Recently, *de novo SCN2A* mutations in autism spectrum disorder (ASD) have been identified. The current study characterizes a *de novo* splice site mutation in *SCN2A* that alters mRNA and protein products.

**Case presentation:**

We describe results from clinical and genetic characterizations of a seven-year-old boy with ASD. Psychiatric interview and gold standard autism diagnostic instruments (ADOS and ADI-R) were used to confirm ASD diagnosis, in addition to performing standardized cognitive and adaptive functioning assessments (Leiter-R and Vineland Adaptive Behavior Scale), and sensory reactivity assessments (Sensory Profile and Sensory Processing Scales). Genetic testing by whole exome sequencing revealed four *de novo* events, including a splice site mutation c.476 + 1G > A in *SCN2A*, a missense mutation (c.2263G > A) causing a p.V755I change in the *TLE1* gene, and two synonymous mutations (c.2943A > G in the *BUB1* gene, and c.1254 T > A in C10orf68 gene). The *de novo SCN2A* splice site mutation produced a stop codon 10 amino acids downstream, possibly resulting in a truncated protein and/or a nonsense-mediated mRNA decay. The participant met new DSM-5 criteria for ASD, presenting with social and communication impairment, repetitive behaviors, and sensory reactivity issues. The participant’s adaptive and cognitive skills fell in the low range of functioning.

**Conclusion:**

This report indicates that a splice site mutation in *SCN2A* might be contributing to the risk of ASD. Describing the specific phenotype associated with *SCN2A* mutations might help to reduce heterogeneity seen in ASD.

## Background

Autism spectrum disorders (ASDs) are a group of neurodevelopmental disorders characterized by social and communication impairments, repetitive behaviors, and sensory reactivity issues [1]. The etiology of ASD is genetically heterogeneous and *de novo* mutations and copy number variations account for 15-20% of ASD cases [2]. Recently, Sanders et al. [14] and Neale et al. [3] reported *de novo SCN2A* mutations (which codes for the alpha subunit of voltage-gated, type II sodium channel) in three cases with ASD, one of which [3] will be described in detail herein.

*SCN2A* is involved in generating action potentials in neurons and muscles and is highly expressed in the brain, specifically in neurons and glia [4]. A recent paper highlights the role of sodium channels in psychiatric disorders and neurological diseases [4]. Sodium-channel disruption, caused by mutations on *SCN2A* and related genes such as *SCN1A* or *SCN9A,* has been associated with ASD, intellectual disability, ataxia and increased sensitivity to pain [5-8]. *SCN2A* mutations have also been associated with various forms of epilepsy [9-12]. A case report documented seizures in a participant with *SCN2A* mutation, along with abnormal visual fixation and poor muscle tone [13]. However, not all patients with *SCN2A* mutations have been reported to have epilepsy [14,15].

In the current study, we characterize a *de novo* splice site mutation in *SCN2A* that alters mRNA. We used gold standard autism diagnostic instruments, structured clinical interview, standardized cognitive testing, and sensory reactivity assessments to probe for diagnostic status, cognitive and adaptive functioning, and sensory reactivity, including hyper-reactivity to stimuli in the environment. Describing phenotypes of *SCN2A* cases will eventually help to reduce the phenotypic heterogeneity seen in individuals with ASD.

## Case presentation

### Methods

The participant was ascertained as part of ongoing IRB-approved genetic research in ASD at the Seaver Autism Center for Research and Treatment at the Icahn School of Medicine at Mount Sinai. Genetic testing included chromosomal microarray analysis (CMA) (Agilent Human CGH 1 × 244A, Agilent Technologies, Santa Clara, CA) and whole exome sequencing (WES). Clinical assessments included: (1) neurological examination conducted by a pediatric neurologist; (2) psychiatric evaluation using the DSM [16] conducted by a board-certified child and adolescent psychiatrist; (3) the Autism Diagnostic Observation Schedule (ADOS) [17]; (4) the Autism Diagnostic Interview Revised (ADI-R); (5) cognitive testing using the Leiter International Performance Scale-Revised [18]; (6) adaptive ratings using the Vineland Adaptive Behavior Scales-II, Survey Edition [19]; and (7) tests of sensory reactivity, including the Caregiver Sensory Profile, a 125-item questionnaire [20] and the Sensory Processing Scales [21]. Magnetic Resonance Imaging (MRI) was conducted to check for structural brain abnormalities (Table 1).

**Table 1:** Domains and measures used in the current study

**Domain**	***Measure***
Genetic testing	
	*WES*
	*CMA*
Clinical assessment	
Neurological examination	*Clinical genetics; height, weight, dysmorphic features*
Autism spectrum disorder (ASD) traits	*ADOS*
	*ADI-R*
Cognitive and adaptive behavior skills	*Leiter-R*
	*Vineland Adaptive Behavior Scale*
Sensory reactivity	*Sensory Profile*
	*Sensory Over-Responsivity Scale*
Brain development	
Structural Imaging	*MRI*

*Abbreviations*: CMA = chromosomal microarray analysis; WES = whole exome sequencing; ADOS = Autism Diagnostic Observation Schedule; ADI-R = Autism Diagnostic Interview Revised; MRI = Magnetic Resonance Imaging.

### Genetic testing

#### Whole exome sequencing

High quality genomic DNA from peripheral blood of the participant and both parents were used for library preparation. A total of 3 ug DNA were sheared separately with a Covaris Acoustic Disruptor (E210) instrument set to time 250 sec, duty cycle 10, intensity 10 and 200 cycles per burst. The size distribution of fragments was centered about 200 bp and verified by Agilent Bioalayzer. NEBNext™ DNA Sample Prep Master Mix Set 1 (New England BioLabs) was used for creating the DNA library. During the adaptor ligation, a six-base sequence index was introduced into the adapter for multiplex sequencing on the Illumina platform.

NimbleGen Seq Cap EZ SR v2 kit (Roche NimbleGen) was used for whole exome library preparation using standard protocol. Prepared libraries were then sequenced with the Hiseq 2000 - V2.5 flowcells. The standard Illumina software was used for de-multiplexing.

Sequencing data was processed with Picard ( http://picard.sourceforge.net/), which utilizes base quality-score recalibration and BWA for mapping reads to hg19. Genetic variants were called using GATK and were annotated by AnnTools ( http://anntools.sourceforge.net/). Potential *de novo* mutation was defined as a heterozygous genotype in the participant and observed reference homozygote genotypes in both parents and in the absence of any other copy of the alternate allele. Each putative *de novo* event was validated in the participant and both parents using the Sanger sequencing method.

#### RT-PCR and cDNA sequencing in lymphoblastoid cell lines

Lymphoblastoid cell lines from the participant and parents were cultured and used to isolate total RNA using the RNeasy Mini kit (Qiagen). SuperScript® II reverse transcriptase and random primers (Life Technology) were used to generate cDNA from the RNA. Different pairs of primer from exon 2 to exon 6 were designed to amplify wildtype and mis-spliced cDNA.

### Clinical assessments

#### Autism spectrum disorder (ASD) traits

The Autism Mental Status Exam (AMSE) was used to structure the direct observation of the subject during the clinical evaluation and was performed by a child and adolescent psychiatrist with expertise in ASD [22]. The ADOS, Module 1, was administered at age three and again at age seven. The ADI-R was conducted with the mother when the participant was three years old [23].

#### Cognitive and adaptive evaluations

The Leiter-R relies on nonverbal instructions for administration, requires minimal motor skills, and measures only nonverbal elements of cognitive functioning [18]. Ratio IQs were calculated using mental age estimates from the cognitive tests and used to provide an estimate of nonverbal IQ. The Vineland Adaptive Behavior Scale was used to measure communication, daily living skills, socialization and motor skills [19].

#### Sensory assessment

The Sensory Profile is a validated instrument, which was standardized using a sample of over 1000 typical developing children [20]. The Sensory Profile is a 125-item questionnaire that has identified sensory differences in over 90% of children and adults with ASD, compared to controls [24-31] and was used to query parents about the frequency (1 = always; 5 = never) of sensory behaviors such as, ‘avoids going barefoot, especially in sand or grass (item 3). The parents also filled out the Sensory Processing Scale (SP Scale) [21,32], which includes 60 items for Sensory Over-Responsivity (SOR) (*These smells bother my child, e.g. soap*) and 30 items for Sensory Under-Responsivity (*Typically my child does not notice e.g. strong odors*). Unlike other scales, the SP Scale reflects sensory processing across all sensory domains (tactile, visual, olfactory, auditory, vestibular, proprioception and gustatory). Parents complete the Inventories by scoring each item as a "1" if the behavior describes their child. Total scores are then computed for each subtype.

## Results

### Whole exome sequencing results

The participant first presented to the Seaver Autism Center for Research and Treatment at Mount Sinai for evaluation at the age of three. At that time, chromosome microarray was performed and no pathogenic alterations were detected. Follow-up testing using whole exome sequencing subsequently identified the *SCN2A* mutation, and the participant returned for another clinical assessment at age seven.

A total of four *de novo* events were identified by whole exome sequencing, including a splice site mutation c.476 + 1G > A in *SCN2A*, a missense mutation (c.2263G > A) causing a p.V755I change in the *TLE1* gene, and two synonymous mutations (c.2943A > G in the *BUB1* gene, and c.1254 T > A in C10orf68 gene). All of the four de novo mutations were heterozygous mutations and none have been reported in the dbSNP or other genomic databases. We validated each mutation and its de novo status by Sanger sequencing in the participant and parental DNA.

The *SCN2A* splice site mutation was in the donor site of intron 4 of the longest isoform (NM_021007) of *SCN2A*. This exon is conserved in two other isoforms (NM_001040142 and NM_001040143) of the gene as reported in the Refseq gene database.

**Figure 1:**
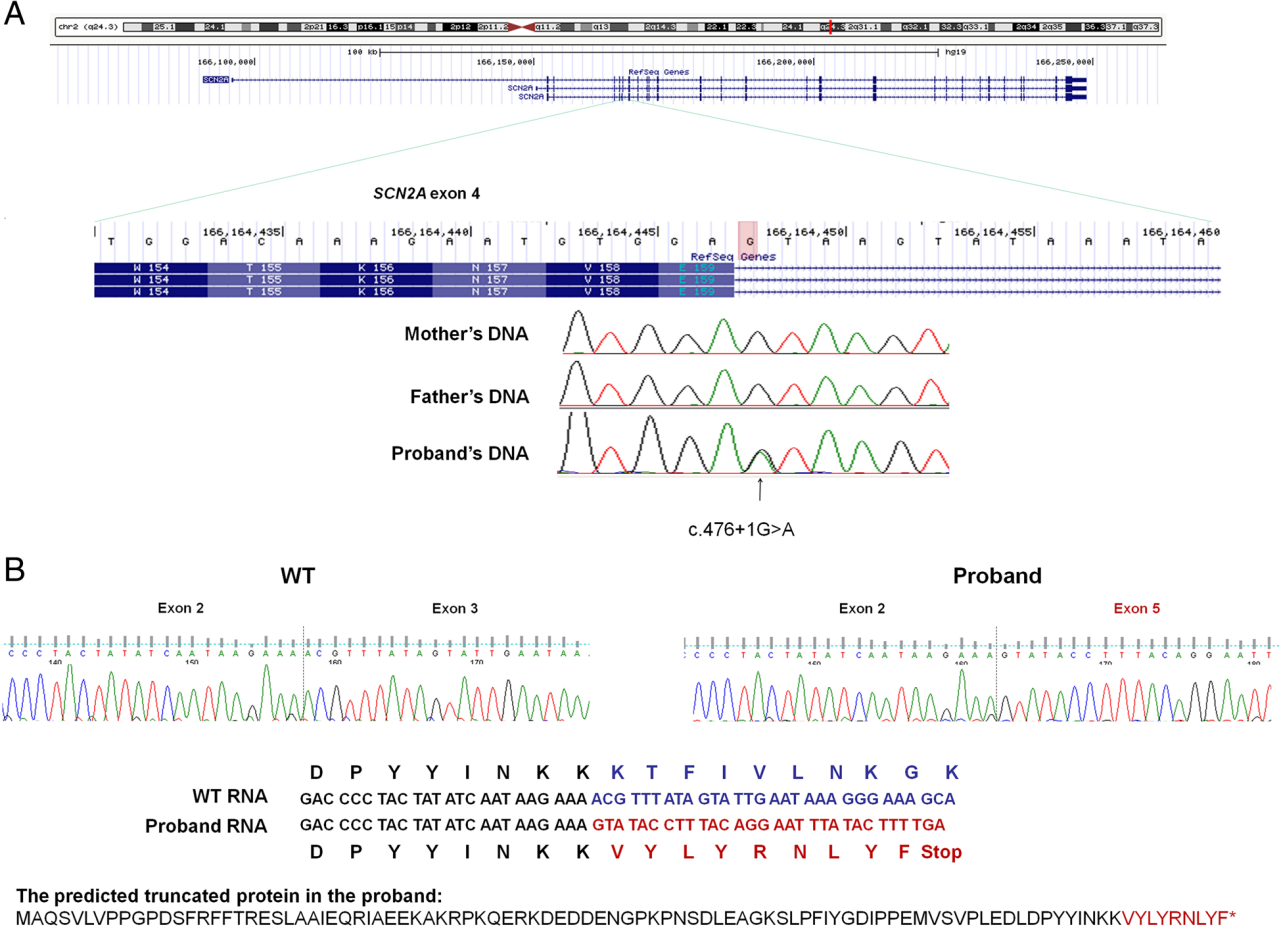
**A *de novo SCN2A *splice mutation in a participant with ASD. (A)** The *de novo* splice mutation c.476 + 1G > A is in the donor site of intron 4, which is conserved over three transcripts of *SCN2A* gene. **(B)** cDNA sequencing in the lymphoblastoid cell lines shows both exon 3 and exon 4 were spliced out in the participant, likely resulting in a truncated short protein.

Since the splice site mutation could affect mRNA splicing, we further examined cDNA of *SCN2A* in cell lines from the participant and parents (see Figure 1). cDNA sequencing showed both exon 3 and exon 4 were spliced out in the participant, while it is expressed normally in the parents. The mis-splicing caused 209 nt deletion in the mRNA and altered the reading frame of the mRNA starting with amino acid 90. It produced a stop codon 10 amino acids downstream, possibly resulting in a truncated protein and/or a nonsense-mediated mRNA decay.

### Clinical assessments

#### Neurological and clinical genetic examinations

The participant’s height was 136 cm at age seven years and nine months (75^th^ percentile), his weight was 32.8 kg (82^nd^ percentile) and his head circumference was 52.2 cm (50^th^ percentile). The participant showed some mild dysmorphic features, including eye asymmetry, fleshy earlobes, and a gap between front teeth. On a cardiovascular exam, the participant had regular heart rate and rhythm, and no murmur was apparent. Motor examination revealed mild hypotonia, but normal strength. Deep tendon reflexes and extensor signs were tested but difficult to elicit. There was no clonus. The participant exhibited a broad-based, abnormal gait with some waddling. He was unsteady standing or walking on uneven surfaces. However there were no abnormal movements. Cranial nerve examination was normal and visual fields were full. Sensory examination elicited symmetrical responses to touch and pain.

#### Psychiatric evaluation

The participant’s parents reported longstanding impairment in social and communicative functioning. His parents reported that his receptive language skills were more advanced than his expressive language. There was no report of delayed or immediate echolalia but there was report of intermittent vocal stereotypy. The participant did not exhibit the capacity for oral motor imitation skills. In the behavioral domain, the participant demonstrated repetitive, compulsive-like behaviors, including repetitive opening and closing of doors. Motor stereotypies were characterized by arm flapping. Encompassing pre-occupations were reported as specific pre-occupations with toys that emit noise.

The participant also had a low frustration tolerance and displayed intermittent tantrum behaviors, which in their severest form included banging his head with his arms and fists. With regard to adaptive functioning, the participant was not consistently bladder trained at night and was not bowel trained during the day. With regard to sensory symptoms, the participant had a notably high pain threshold, and he did not tolerate certain types of touch (e.g. getting a haircut). The participant was described as a picky eater.

As for medical history, the parents reported no history of seizures. However, in the first year of life there were frequent episodes of becoming "stone faced" and "limp". Pregnancy and birth were normal. Parents were both in their mid-thirties when the participant was born. There was no family history of ASD, language delay, intellectual disability or Attention Deficit/Hyperactivity Disorder. Family history was positive for Obsessive Compulsive Disorder.

Regarding developmental history, the participant had motor delays; he did not sit or crawl and only began walking at approximately two years of age. The participant had longstanding language impairment and had only begun using single words at age 4, after beginning Applied Behavioral Analysis (ABA). There was no report of loss of skills. As for educational history the participant was referred for early intervention services at 18 months due to concerns about motor and language delays at which time he began receiving occupational therapy (OT) and physical therapy (PT).

#### Autism spectrum disorder diagnostic test

##### Mental status exam

The participant showed significant social impairment and was unable to engage in joint attention or social reciprocity on examination. Although he was able to make fleeting eye contact, he was unable to point or follow pointing. Further, the participant showed motor stereotypies such as hand flapping and engaged in compulsive opening and closing of doors.

##### ADOS

Overall, the participant’s scores were consistent with a classification of autism in the areas of communication, reciprocal social interactions and repetitive behaviors, and in total at age three and at age seven. As for communication, at age seven the participant directed vocalizations towards the examiner infrequently and used mostly single words, along with an occasional phrase for the purpose of requesting (e.g. "Go bathroom"). The intonation of his speech tended either to be flat or to take on a "sing-song" quality. With respect to nonverbal communication, he did not point or use any other common gestures throughout the assessment (e.g., head nodding, shrugging).

As for the social domain, the participant demonstrated several emerging social skills, including the ability to respond to his name as well as bids for joint attention. However, eye contact and social responsiveness were inconsistent throughout the observation.

Regarding repetitive and stereotyped behaviors, the participant demonstrated sensory interests and hand mannerisms, such as touching the length of his index finger to his mouth and flapping his hands, respectively.

##### ADI-R

The participant met criteria for autism in all three diagnostic domains (Qualitative Abnormalities in Reciprocal Social Interaction, Qualitative Abnormalities in Communication, and Restricted, Repetitive and Stereotyped Patterns of Behavior) as reported by his mother.

According to his mother’s report, the participant was generally unresponsive to unfamiliar people, but he was responsive to familiar individuals, smiled, and displayed a range of appropriate facial expressions. Language was described as limited to two-word phrases, and beyond shaking his head or occasionally nodding, he did not use nonverbal gestures. Finally, the participant displayed excessive interest in books, prompting his parents to carry a book with them everywhere; he was reported to line up his toys, tended to focus on part of his toys rather than the whole toy, and was particularly drawn to visual reflections.

### Cognitive and adaptive evaluations

#### Leiter international performance subscales

At age three years and nine months, the participant’s Full Scale IQ was estimated to be 53, which falls into the extremely low range of functioning. At age seven years and nine months, the estimated Full Scale IQ was 45, which falls in the extremely low range as well. This apparent decline in IQ between the ages of three and seven is unlikely to be related to loss of skills but instead due to the lack of appropriate gains as compared to typically developing peers. The participant had difficulty attending to tasks and could not complete all subtests. A relative strength was the participant’s ability to match basic visual-perceptual stimuli (see Table 2).

**Table 2:** Leiter International Performance Scale scores at ages three and seven

***Subtest***	**Scaled score at age 3 years 9 month**	**Scales score at age 7 years and 9 month**
***Visualization***		
*Figure ground*	4	1
*Matching*	5	1
*Picture context*	4	-
*Paper folding*	-	2
*Form completion*	5	1
***Reasoning***		
*Classification*	2	-
*Sequential order*	3	5
*Design analogies*	-	5
*Repeated patterns*	4	1

#### Adaptive behavior

The participant had an overall adaptive behavior composite score and sub scores in the low to moderately low range (see Table 3).

**Table 3:** Summary of Vineland Adaptive Behavior Scale results

***Vineland Adaptive Behavior Scale***	***SCN2A *** **case**	**Compared to normative scores**
***Adaptive behavior composite***	64	1^st^ percentile, low range
***Communication***	35	8^th^ percentile, low range
***Daily living skills***	25	1^st^ percentile, low range
***Socialization***	30	3^rd^ percentile, low range
***Motor skills***	17	1^st^ percentile, low range

### Sensory assessment

#### Sensory profile

The participant presented with sensory reactivity abnormalities as reported by his parents on the widely used Sensory Profile. Definite differences were reported (2 standard deviations above the mean) on oral, tactile, multisensory and vestibular processing. Probable differences (one standard deviation above from mean) were reported on auditory processing (see Table 4). In terms of sensory modulation, definite differences were shown on sensory processing related to endurance/tone, modulation of movement affecting activity level, and modulation of sensory input affecting emotional response. On the Factors of the Sensory Profile, the participant showed definite differences on all factors other than sensory sensitivity, which was in the typical range. Responses on all other factors including sensory seeking, poor registration, low endurance, fine motor, sedentary and distractibility showed definite differences as compared to typically developing children. Taken together the participant shows severe sensory reactivity issues.

**Table 4:** Summary of the participant’s sensory profile scores

***Sensory processing***	***SCN2A *** **case**	**Typical performance**	**Probable difference**	**Definite difference**
***Oral sensory processing***	25	55-48	47-45	44-11
***Touch processing***	59	90-73	72-65	64-18
***Multisensory processing***	19	35-27	26-24	23- 7
***Vestibular processing***	43	55-48	47-45	44-11
***Visual processing***	26	45-32	31-27	26-9
***Auditory processing***	26	40-30	29-26	25-8

The classifications of typical, probable and definite differences were based on the performance of children without disabilities (n = 1,037, see Dunn, 1997). Probable differences are characterized as being over 2 standard deviations away from norm and definite differences as being over 3 standard deviations away from norm.

#### Sensory over-responsivity inventory

The parents endorsed 35% of the items (21 out of 60) on the Sensory Over-Responsivity Scale (see Table 5). The participant’s score of 21 fell into the category of being sensory over-responsive. Specifically, tactile accessories (scarf, hat etc.), fuzzy or furry textures, wool clothes, washing or wiping face, cutting nails, having hair cut, and light stroking of the skin bother the participant. In terms of vision, brightly color material, fluorescent light, visually cluttered environments, and busy pictures bothered the participant. In terms of sound, small motor noises, restaurants and large gatherings bothered the participant. In addition, the parents endorsed 14 out of 31 items on the Sensory Under Responsivity Inventory. Parents reported that the participant responds less than others to bruises or cuts, hurting himself, dirtying himself, and bumping into things. In addition, the participant does not notice when his hands or face are messy/dirty, when he needs to use the toilet, or if an object cames close to his eyes.

**Table 5:** Participants scores on the sensory over- responsivity scale

***Sensory processing***	***SCN2A *** **score**	**Typical performance**	**Children with sensory processing difficulties**
***Sensory over-reactivity***	21	4 ± 4.7	18.5 ± 13.9

Scores for typically developing participant’s and participants with sensory difficulties were taken from Schoen, et al. 2008 [32].

### Brain development

#### MRI

A brain MRI was performed using a Siemens Allegra 3 T head-only scanner. The research protocol on the diagnostic images showed no evidence of infra or supratentorial masses. The lateral ventricles appeared normal. No midline shift was appreciated and the ventricles and sulci were within normal limits. No white matter abnormalities were appreciated. The flow voids in the vessels appeared grossly within normal limits. The overall impression showed a normal research protocol of the brain.

## Conclusions

The current study describes a 7-year-old boy with a *de novo SCN2A* splice site mutation (c.476 + 1G > A). Although this particular mutation has not been reported before, it could be considered as contributing to risk of ASD by causing abnormal gene splicing, leading to significantly shortened protein product and/or an abnormal message that is subject to nonsense-mediated mRNA decay. The participant also presented with three other *de novo* events, one missense mutation in *TLE1* gene, and two synonymous mutations in *BUB1* gene. While the four de novo mutations support the notion of multiple hits in ASD, the *de novo SCN2A* splice site mutation, a recently identified ASD gene, is likely to be driving the severity of symptoms. The participant presented with ASD as diagnosed using psychiatric evaluation and confirmed by the ADOS and ADI-R. The participant showed social and communication problems, repetitive behaviors and sensory over- and under-reactivity. Neurological and clinical genetic examinations revealed hypotonia, abnormal gait and fine motor coordination, and mild dysmorphic features. Further, the participant’s adaptive and cognitive skills fell in the low range of functioning. No structural brain abnormalities were evident on MRI, which is consistent with past reviews of participants with sodium channel disruptions [11].

Interestingly, the participant does not have any history of seizures. Participants with *SCN2A* mutations have been identified with seizures and various forms of epilepsy [15]. However, in the current case, no seizures were observed. Although electroencephalography was attempted, the participant’s sensory reactivity issues prevented any usable data from being recorded. The absence of seizures is consistent with a recent study by Sanders et al. (2012), which reported two *de novo SCN2A* cases that did not show any history of seizures [14]. However, in our study it was noted that there were frequent episodes of becoming "stone faced" and "limp" in the first year of life that may have been indicative of seizure activity. On the other hand, since *SCN2A* mutations are linked to various forms of epilepsy, a late onset cannot be ruled out either [33].

This participant demonstrated with significant sensory reactivity issues. Most sensory reactivity differences were reported on oral, tactile, multisensory, vestibular, and visual processing followed, less markedly, by auditory processing. The participant fell into the ‘sensation seeker’ and ‘poor registration’ category. Further, the participant seemed to be overwhelmed by some aspects of the environment such as touch (washing face, wool clothes), while being under-responsive to other aspects of the environment (bruises, cuts). Pain related perception is processed via nociceptors (e.g., unmyelinated C fibres) whereas light touch is processed by cutaneous receptors (e.g. Meissner’s corpuscles or Merkel discs) [34]. Examining sensory reactivity in cases with ASD and *SCN2A* mutations is important because comorbid sensory symptoms often compromise an individual’s and family’s quality of life [35]. Sensory over-reactivity has also been linked to high rates of anxiety in individuals with ASD [33,36,37].

Future research is needed to confirm the phenotypes observed in the current case report in more cases with *SCN2A* mutations. Identifying a robust phenotype for *SCN2A* cases will help to reduce the phenotypic heterogeneity seen in individuals with ASD.

## Consent

Written informed consent was obtained from the parents of the patients.

## Competing interests

The authors of this manuscript report no competing interests.

## Authors’ contributions

JDB, AK, DG, ATW, DH and TT participated in the design of the study. LS, LB, ATW and SS coordinated the study and carried out the data collection and MRI acquisition. JC-L, DH and DG carried out the clinical assessments. GC carried out chromosomal microarray analysis and whole exome sequencing. TT drafted the manuscript and all authors read and approved the final manuscript.

## Pre-publication history

The pre-publication history for this paper can be accessed here:

http://www.biomedcentral.com/1471-2350/15/35/prepub
